# D11-Mediated Inhibition of Protein Kinase CK2 Impairs HIF-1α-Mediated Signaling in Human Glioblastoma Cells

**DOI:** 10.3390/ph10010005

**Published:** 2017-01-01

**Authors:** Susanne Schaefer, Tina H. Svenstrup, Mette Fischer, Barbara Guerra

**Affiliations:** Department of Biochemistry and Molecular Biology, University of Southern Denmark, 5230 Odense, Denmark; sschaefer@bmb.sdu.dk (S.S.); thl@bmb.sdu.dk (T.H.S.); mettefischer@outlook.com (M.F.)

**Keywords:** CK2, D11, HIF-1α, glioblastoma cells, gene expression profiling

## Abstract

Compelling evidence indicates that protein kinase CK2 plays an important role in many steps of cancer initiation and progression, therefore, the development of effective and cell-permeable inhibitors targeting this kinase has become an important objective for the treatment of a variety of cancer types including glioblastoma. We have recently identified 1,3-dichloro-6-[(*E*)-((4-methoxyphenyl)imino)methyl]dibenzo(b,d)furan-2,7-diol (D11) as a potent and selective inhibitor of protein kinase CK2. In this study, we have further characterized this compound and demonstrated that it suppresses CK2 kinase activity by mixed type inhibition (K_I_ 7.7 nM, K_I_′ 42 nM). Incubation of glioblastoma cells with D11 induces cell death and upon hypoxia the compound leads to HIF-1α destabilization. The analysis of differential mRNA expression related to human hypoxia signaling pathway revealed that D11-mediated inhibition of CK2 caused strong down-regulation of genes associated with the hypoxia response including *ANGPTL4*, *CA9*, *IGFBP3*, *MMP9*, *SLC2A1* and *VEGFA*. Taken together, the results reported here support the notion that including D11 in future treatment regimens might turn out to be a promising strategy to target tumor hypoxia to overcome resistance to radio- and chemotherapy.

## 1. Introduction

Glioblastoma is the most common primary tumor of the central nervous system and one of the most lethal types of human cancer. Patients with newly diagnosed glioblastoma have a median survival of approximately one year and respond poorly to all treatment modalities [1,2]. A comprehensive characterization of over 200 samples from patients diagnosed with glioblastoma has provided a detailed map of the many genomic and transcriptional alterations that occur in this type of malignancy and that contribute to its aggressive character [3,4,5]. Overexpression of the epidermal growth factor receptor (EGFR), which is linked to aberrant phosphoinositide 3-kinase (PI3K/AKT) pathway, is among the most frequent aberrations. However, cancer genomic studies have revealed that multiple components of the aforementioned pathway are frequently targeted by germline or somatic mutations contributing to tumor resistance to both radiotherapy and chemotherapy [5]. In this respect, glioblastoma has one of the highest incidence rates of phosphatase and tensin homolog (*PTEN*) mutation that has been strongly associated with a selective advantage for tumor expansion [6]. Loss of PTEN expression results in hyperactivation of the pro-survival PI3K/AKT pathway and increased resistance to apoptosis [7].

All tumors require active angiogenesis for expansion and glioblastoma is a highly vascularized type of cancer exhibiting increased expression of many pro-angiogenic genes such as vascular endothelial growth factor (*VEGF*) and fibroblast growth factor (*FGF*) [8]. Despite the extended vascularization, glioblastoma is highly heterogeneous and dominated by areas with low oxygen supply and compromised vascular integrity [9].

One of the major events in hypoxia adaptation is the induction of the hypoxia inducible factor 1 (HIF-1α) a transcription factor that regulates multiple intracellular processes including glycolysis, angiogenesis, immortalization, tissue invasion and metastasis, genetic instability and cell death (reviewed in [10]). From a therapeutic standpoint, hypoxia, genomic and tumor-tissue heterogeneity are a major clinical hurdle emphasizing the importance to set up tailored therapeutic strategies able to tackle the heterogeneous nature of brain cancer. Up-regulation of HIF-1α leads to a rapid response in hypoxic cells. Thus, identification of compounds able to block HIF-1α or its target genes for inhibiting tumor growth has become a top priority in many research laboratories.

We have recently identified 1,3-dichloro-6-[(*E*)-((4-methoxyphenyl)imino)methyl]dibenzo-(b,d)furan-2,7-diol (hereafter referred to as D11) as a potent and selective inhibitor of protein kinase CK2 [11,12]. CK2 is a highly conserved and constitutively active serine/threonine protein kinase whose expression levels have been found invariably elevated in highly proliferating cells. CK2 is implicated in multiple cellular functions as well as in cell transformation and tumorigenesis [13,14,15,16,17]. Recent data from our laboratory have demonstrated that D11 treatment induces apoptosis in brain and pancreatic cancer cells through a mechanism involving down-regulation of the PI3K/AKT and NF-κB signaling cascades [12]. Interestingly, inhibition of CK2 has been shown to decrease HIF-1α activity under hypoxia through elevated p53 expression levels [18].

Since the high incidence of PTEN mutation observed in glioblastoma cells has been strongly associated with stimulation of HIF-1α-mediated gene expression and increased apoptotic resistance [6], we investigated whether D11-mediated cell death induction was accompanied by destabilization and/or inhibition of HIF-1α transcription factor and, if so, how does D11 influence the expression of genes regulated by HIF-1α under hypoxic conditions. Results shown in this study suggest that D11 may exert anti-tumor activity by negatively affecting cellular pathways that are normally up-regulated under low oxygen tension and warrant in vivo studies for endorsing its efficacy in the treatment of multi-drug resistant human cancers.

## 2. Results

### 2.1. D11 is A CK2 Inhibitor That Leads to Cell Death in Glioblastoma Cells

D11 was identified employing the catalytic α-subunit of protein kinase CK2 following a screening of a small-molecule compound library of the Diversity Set III under the drug discovery and therapeutic program (DTP) of the National Cancer Institute (NCI, Rockville, MD, USA, [11]). Kinetic measurements revealed that this compound inhibits the catalytic activity of both CK2α and CK2 holoenzyme in the low nM range [11]. Furthermore, the screening of 354 protein kinases revealed that D11 is a selective inhibitor of CK2. By setting the threshold for inhibition to >98%, only three protein kinases were found inhibited to an extent observed with CK2 [11].

**Figure 1:**
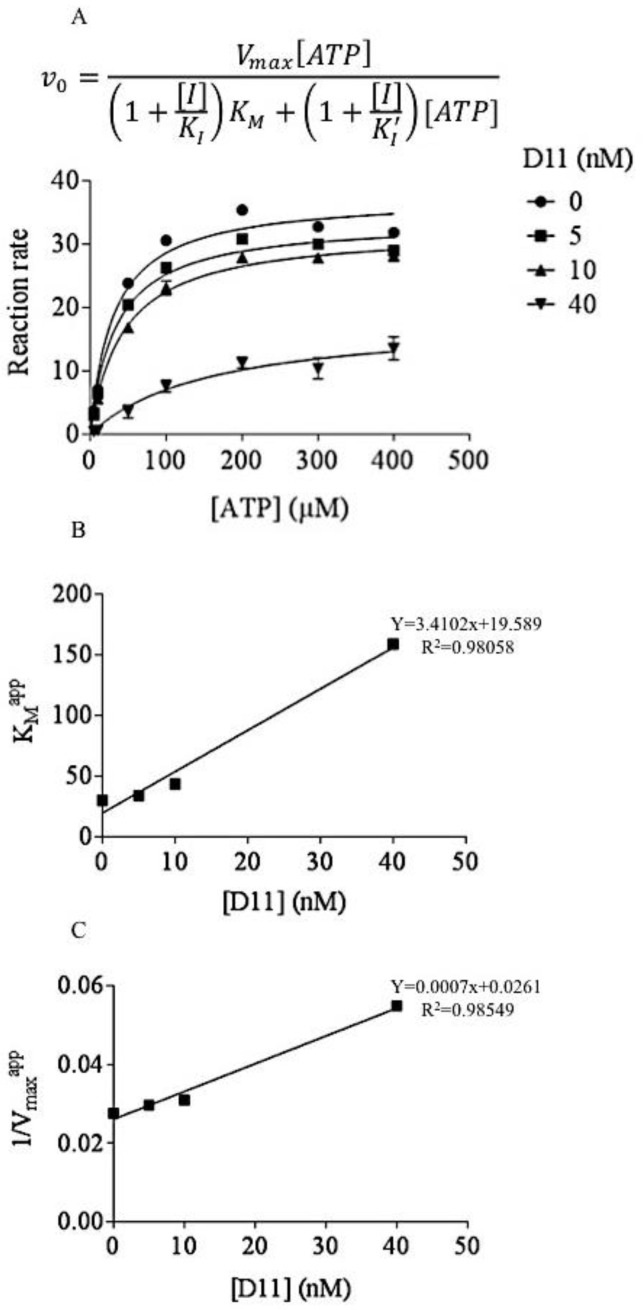
Kinetic mechanisms of CK2 inhibition. (**A**) Michaelis-Menten curves showing how velocity varies over a range of substrate concentrations for three different concentrations of inhibitor. The velocity equation relative to the hyperbolic curves of the graph for mixed inhibition is shown above the plots. I: inhibitor (D11), K_I_: dissociation constant for binding of inhibitor to free enzyme, K_I_′: dissociation constant for binding of inhibitor to substrate-bound enzyme. (**B**) Apparent K_M_ (K_M_^app^) re-plotted against varying concentrations of inhibitor (used for the calculation of K_I_). (**C**) Reciprocal apparent V_max_ (1/V_max_^app^) re-plotted against varying concentrations of inhibitor (employed for the calculation of K_I_′). The reaction rate is expressed in pmol/min/ng. Experiments were repeated three times.

In this study, we carried out experiments in order to further characterize the type of inhibition exerted by D11 on CK2. Interestingly, increasing concentrations of co-substrate (i.e., ATP) tested with three different concentrations of inhibitor, respectively, resulted in decreased apparent maximum velocity (V^app^_max_) and increased apparent K_M_ (K^app^_M_) values suggesting a linear mixed-type inhibition against ATP as can be seen from the Michaelis-Menten plots (Figure 1A). Non-linear least-squares fits of the curves in Figure 1A to rectangular hyperbolas were used to determine the apparent K_M_ and V_max_ values resulting from the effect of different concentrations of inhibitor. From the estimated apparent K_M_ and V_max_ values we created re-plots for the determination of K_I_ (dissociation constant for binding of D11 to the free enzyme, 7.7 nM, Figure 1B) and K_I_′ (dissociation constant for binding of D11 to the enzyme-substrate complex, 42 nM, Figure 1C).

**Figure 2:**
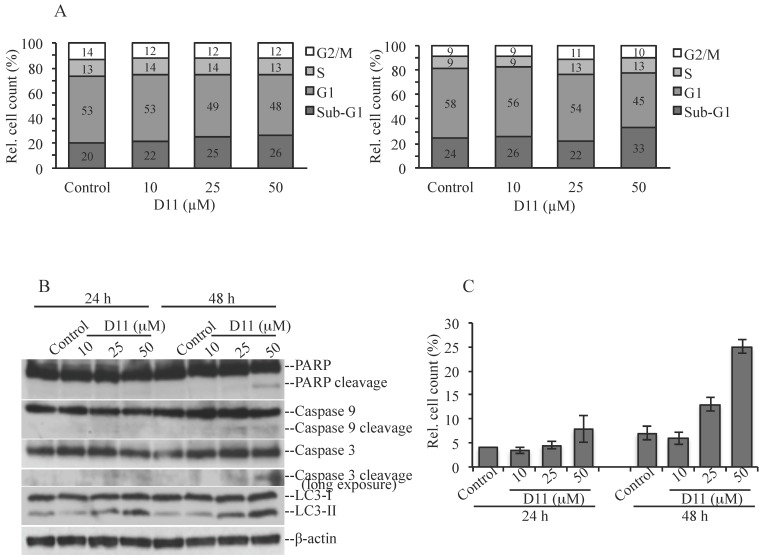
Anti-proliferative effects of D11 in human glioblastoma cells. (**A**) Flow cytometry analysis of cells vehicle-treated (Control, 0.1% DMSO) or incubated with increasing concentrations of D11 for 24 h (left bar graph) and 48 h (right bar graph), respectively. The relative amount of cells in the various phases of the cell cycle is shown in percentage. (**B**) Cells were treated as described above. Whole cell lysate was analyzed by western blot employing antibodies directed against the indicated proteins. β-actin detection was carried out as control for equal loading. (**C**) Flow cytometry analysis of cells treated as described in (**A**) stained with acridine orange for the determination of autophagic vacuoles formation. Number of red-positive cells is expressed in percentage. Experiments were performed three times obtaining similar results.

We previously showed that viability of the human glioblastoma cell line U-87 MG decreased by 50% following treatment with 25 μM D11 for 24 h [12]. Here, we carried out a cell cycle analysis by flow cytometry and asked the question whether D11 treatment induced cell death (Figure 2). Cells were treated with increasing concentrations of D11 for 24 h (Figure 2A, left bar graph) and 48 h (Figure 2A, right bar graph), respectively. As compared to control experiments, cells were not significantly affected by treatment with 50 μM D11 for 24 h (Figure 2A, left bar graph). However, incubation of cells with 50 μM D11 for 48 h was accompanied by a modest increase in the percentage of cells in sub-G1 phase indicative of cells that have undergone DNA degradation (Figure 2A, right bar graph). Next, Western blot analysis was carried out in order to determine the type of cell death. As shown in Figure 2B, treatment with 50 μM D11 for 48 h was necessary for inducing cleavage of full-length PARP, caspase 9 and caspase 3. However, a shorter incubation time (i.e., 24 h) was sufficient to induce increased LC3-II signal as compared to control cells suggesting induction of autophagy, in a time and concentration-dependent manner. The staining of cells with acridine orange (Figure 2C), a vital dye, which accumulates in acidic compartments emitting red fluorescence indicative of autophagic vacuoles formation, further confirmed results reported above.

### 2.2. D11 Treatment Abolishes HIF-1α Transcriptional Activity and Results in Increased Activation of Autophagy under Hypoxia in Glioblastoma Cells

**Figure 3:**
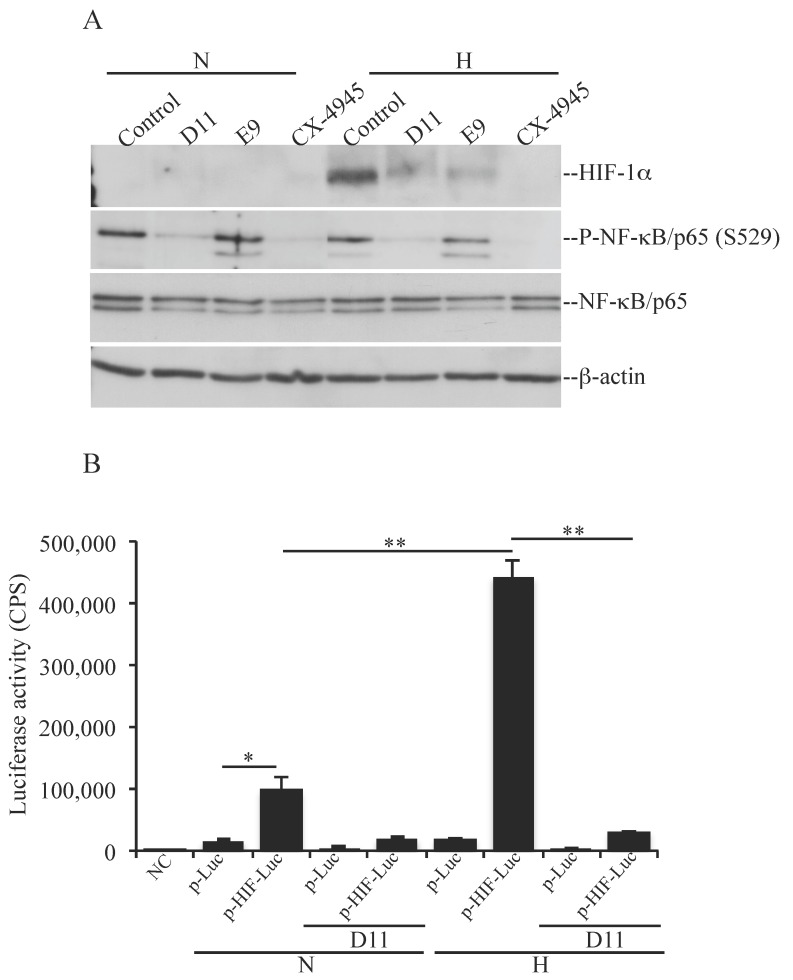
Stabilization and transcriptional activity of HIF-1α under hypoxia is impaired in cells treated with D11. (**A**) U-87 MG cells were treated with vehicle (Control), 50 μM D11, 50 μM E9 or 20 μM CX-4945 for 24 h under normoxia (N) and hypoxia (H), respectively. Whole cell lysate from cells treated as shown in the figure was subjected to western blot analysis and the expression and phosphorylation levels of the indicated proteins were analyzed. β-actin detection was used as loading control. Experiments were performed three times obtaining similar results. (**B**) Cells were transfected with a control plasmid (p-Luc) or a plasmid containing HIF-1α response element (p-HIF-Luc). 24 h from transfection, cells were added vehicle (0.1% DMSO) or 50 μM D11 and incubated under normoxic (N) or hypoxic (H) condition for the subsequent 24 h. HIF-1α transcriptional activity was analyzed employing a luciferase reporter gene assay. HIF-1α activity was measured testing the ability of the transcription factor to bind the HIF-1α response element that controls the expression of the luciferase reporter gene. HIF-1α transcription activity is expressed in counts/s (CPS). Experiments were repeated twice in triplicates obtaining similar results. * *p* < 0.005, ** *p* < 0.0001.

HIF-1 is a heterodimeric transcription factor consisting of two subunits; i.e., HIF-1β, which is constitutively expressed, and HIF-1α whose expression is regulated by oxygen levels. Stabilization of HIF-1α occurs under hypoxic conditions and leads to translocation into the nucleus where it induces the transcription of numerous genes as part to the hypoxia response (reviewed in [10]). Tumor suppressor genes encoding proteins such as the Von Hippel-Lindau (VHL) and PTEN have been reported to inhibit HIF-1α function. In particular, PTEN has been demonstrated to inhibit HIF-1α stabilization by antagonizing the PI3K pathway [6,19,20]. As U-87 MG cells contain mutant PTEN [21], we asked whether D11-mediated inhibition of CK2 resulted in HIF-1α destabilization and if this event had implications for the activation of cell death. To accomplish this, we compared the levels of expression of HIF-1α in cells treated with vehicle (0.1% DMSO), D11 or other known CK2 inhibitors under normoxia and hypoxia, respectively (Figure 3A). Under hypoxic conditions, U-87 MG cells showed induction of HIF-1α. Moreover, treatment with the indicated compounds resulted in inhibition of endogenous CK2 to various extents as indicated by decreased phosphorylation of NF-κB/p65 at S529 a known CK2 phosphorylation site [22]. Results shown in Figure 3A indicate that cell incubation with D11, E9 or CX-4945 resulted in destabilization of HIF-1α indicated by the reduced HIF-1α protein band intensity as compared to control experiments. Next, we analyzed whether D11 affected HIF-1α transcriptional activity employing a Luciferase reporter assay (Figure 3B).

**Figure 4:**
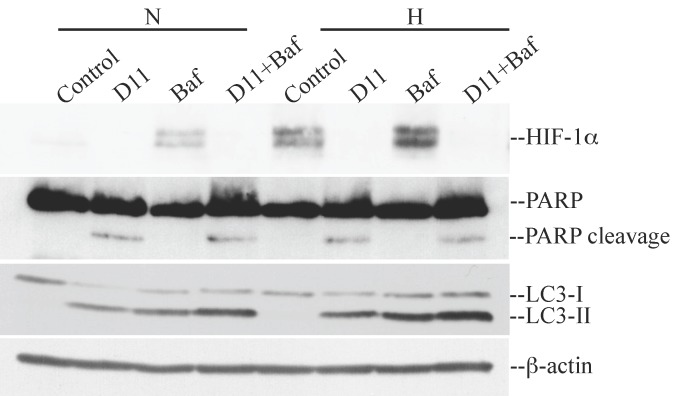
D11-mediated destabilization of HIF-1α under hypoxia is accompanied by higher levels of autophagy. Cells were incubated with 0.1% DMSO, 50 μM D11 alone or in combination with 100 nM bafilomycin A_1_ (Baf) under normoxia and hypoxia, respectively. Cells were treated with D11 for 24 h while bafilomycin A_1_ was added in the last 6 h of incubation time. Proteins were visualized by probing the western blot membranes with antibodies against the indicated proteins.

Cells were transfected with p-HIF1-Luc reporter vector able to express firefly luciferase under the control of a HIF-1-response element (p-HIF-Luc). As a negative control, cells were transfected with a vector lacking the specific response element (p-Luc). The transcription activity of HIF-1α increased dramatically under hypoxic conditions (i.e., 443128 CPS) as compared to its activity under normoxia (i.e., 98746 CPS). Upon treatment with 50 μM D11 for 24 h, luciferase activity induced by HIF-1α stabilization was drastically reduced (i.e., 31917 CPS) under hypoxia supporting the notion that D11 impairs the transcriptional activity of HIF-1α. In order to determine whether destabilization of HIF-1α had an influence on cell death, cells were incubated with D11 under normoxia and hypoxia, respectively. As shown in Figure 4, the exposure of cells to hypoxia did not result in higher PARP cleavage, however, it resulted in higher levels of autophagy induction as compared to cells grown under normoxia. Experiments carried out in the absence or presence of bafilomycin A_1_, which blocks fusion of autophagosomes with lysosomes, revealed that D11 treatment leads to enhanced autophagic flux indicated by a further increase in LC3-II levels in the presence of bafilomycin A_1_.

### 2.3. Cell Incubation with D11 Results in Altered Gene Expression Profile Induced by Hypoxia

**Figure 5:**
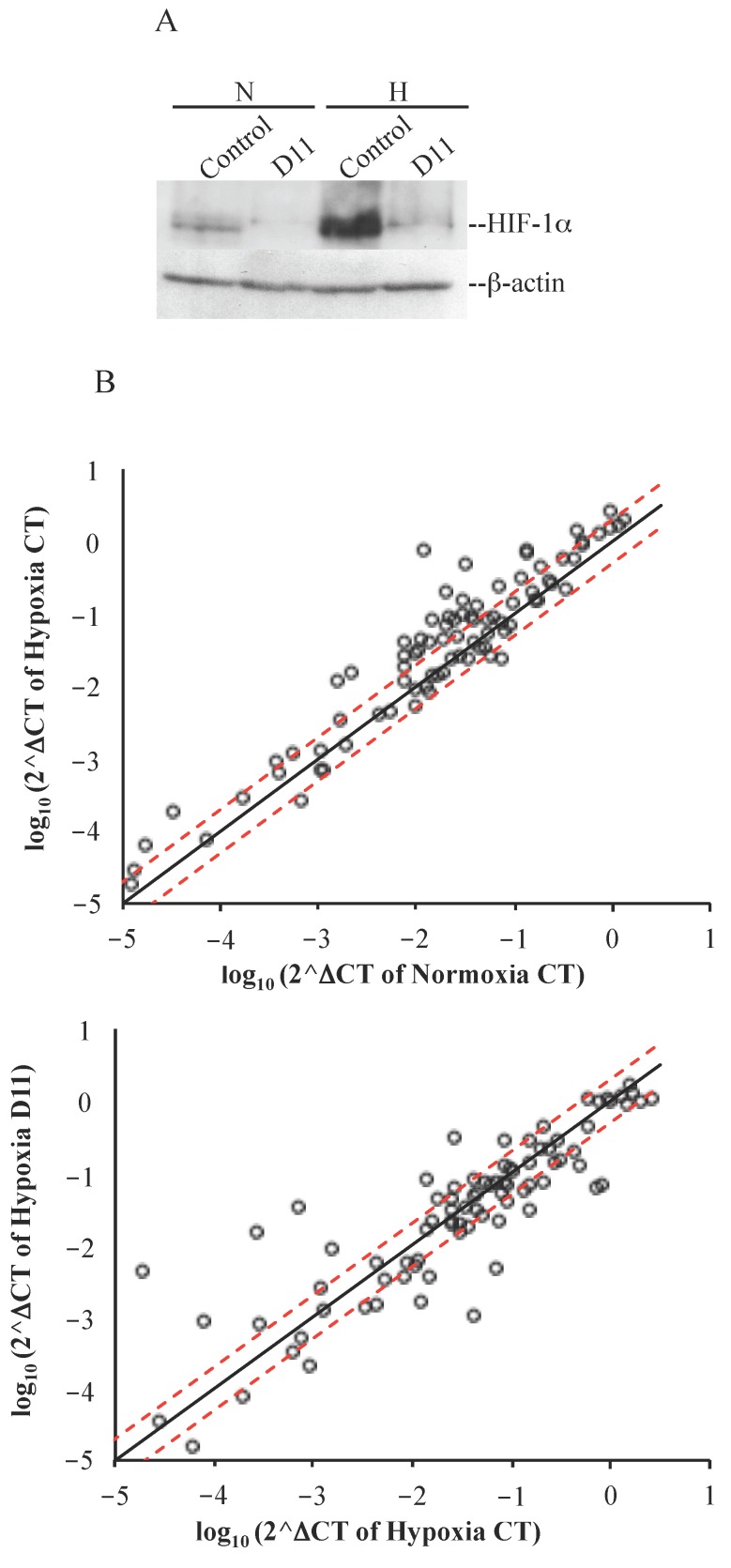
Genes differentially expressed in response to hypoxia and D11 treatment. (**A**) Western blot analysis of whole cell lysate from cells incubated with 0.1% DMSO or 50 μM D11 for 24 h under normoxia and hypoxia, respectively. Western blot membranes were employed for the detection of HIF-1α and β-actin expression levels, respectively. (**B**) Scatter plot of changes of expression of genes in glioblastoma cells. Upper panel: changes of gene expression between cells incubated under normoxia (control) and cells incubated under hypoxia for 24 h. Lower panel: changes of gene expression in the presence or absence of D11 under hypoxic conditions. The fold regulation cut-off (red dashed line) was set on 2.

Stabilization of HIF-1α in the absence of oxygen stimulates the expression of numerous hypoxia-response genes that promote the survival of cancer cells in an unfavorable environment. In order to investigate differential mRNA expression in glioblastoma cells resulting from D11 treatment under hypoxic conditions, we analyzed the expression of 84 genes that respond to low oxygen levels using the human hypoxia-signaling pathway RT^2^ Profiler PCR array. Cells were grown under normoxic or hypoxic conditions for 24 h and incubated either with vehicle (0.1% DMSO) or 50 μM D11 for the same length of time. Based on scatter plot analysis (Figure 5B, upper plot), a number of known HIF-1α-target genes were found up-regulated in control cells in response to hypoxia (CT_H_ vs. CT_N_, fold-change ≥ 2, Table 1). In particular, the strongest up-regulation was observed in the case of angiopoietin-like 4 (*ANGPTL4*), Bcl2/adenovirus E1B 19 kDa interacting protein 3 (*BNIP3*), carbonic anhydrase IX (*CA9*), DNA-damage-inducible transcript 4 (*DDIT4*), coagulation factor III (*F3*), insulin-like growth factor binding protein 3 (*IGFBP3*), lysyl oxidase (*LOX*), matrix metallopeptidase 9 (*MMP9*), N-myc downstream regulated 1 (*NDRG1*), solute carrier family 2 member 1 (*SLC2A1*) and vascular endothelial growth factor A (*VEGFA*). In contrast, when we analyzed the genes that showed differential expression under hypoxic conditions and looked at their up- or down-regulation in response to D11 treatment (i.e., D11_H_ vs. CT_H_), we noticed that most of them failed to become up-regulated or appeared to be partially down-regulated (Figure 5B, lower plot and Table 1). However, strong down-regulation was observed in the case of *F3*, 6-phosphofructo-2-kinase/fructose-2,6-bisphosphatase 4 (*PFKFB4*), solute carrier family 16 member 3 (*SLC16A3*) and *VEGFA* (Table 1). HIF-1α protein expression levels increased under hypoxic conditions (Figure 5A). Conversely, consisting with data reported earlier [23,24], up-regulation of HIF-1α was not accompanied by a change in its mRNA levels in cells exposed to hypoxia as compared to cells incubated under normoxia. Interestingly, D11 treatment resulted in decreased expression of HIF-1α protein but not mRNA levels under hypoxia (results not shown) suggesting alternative D11-mediated mechanisms of regulation of HIF-1α.

## 3. Discussion

Hypoxia is a critical condition for many types of cancers because it signals adaptation of cells to an anaerobic environment achieved by induction of gene expression. Limited oxygen diffusion is frequently observed in the glioma microenvironment and it has been associated with poor prognosis, increased metastasis, angiogenesis and resistance to radio- and chemotherapy. The stabilization of HIF-1α transcription factor is one of the most critical events occurring under hypoxia, as it is the primary factor responsible for many of the effects observed in aggressive tumors. Hence, it is not surprising that HIF-1α has become an important target for drug development in recent years as blocking HIF-1α activity would help starve growing tumors of oxygen and nutrients.

Our in vitro data show that treatment of cells with D11, a small-molecule inhibitor of protein kinase CK2, results in destabilization of HIF-1α protein under hypoxic conditions. Similar results were also reported in the case of other CK2 inhibitors (i.e., E9, CX-4945 and quercetin, [25]). It has been demonstrated that HIF-1α mRNA levels do not vary under hypoxic conditions [23,24]. Accordingly, the analysis of gene expression data did not reveal any significant differences in the expression of *HIF-1α* in cells growing under normoxia or exposed to hypoxia in the absence and presence of D11, respectively, suggesting that regulation of HIF-1α expression occurs at the post-translational level. Apart from CK2 inhibitors, other compounds can induce HIF-1α destabilization. The small molecule inhibitor YC-1 [3-(-5′-hydroxymethyl-2′-uryl)-1-benzylindazole] was shown to reduce HIF-1α levels and xenograft growth of various human tumors through mechanisms yet to be elucidated [26]. Under hypoxic conditions, HIF-1α stability is dependent on its interaction with the chaperone HSP-90 and cell incubation with the HSP-90 inhibitor 17-allylamino-17-demethoxygeldanamycin (17-AAG) has been shown to induce HIF-1α degradation in a VHL-independent manner [27,28,29].

The activity of chaperone proteins is dependent on their interaction with co-chaperone proteins and co-activators [30]. Compelling evidence has indicated that CK2-mediated phosphorylation of the co-chaperone CDC37 is essential for stabilization of HSP-90-CDC37 heterocomplex and its interaction with client protein kinases (reviewed in [31]). Hence, HIF-1α degradation observed in cells incubated with D11 under hypoxia might result from disruption of HSP-90-CDC37 interaction as cell treatment with this inhibitor has been reported to reduce CDC37 phosphorylation [11] and destabilize HSP-90-CDC37 heterocomplex [12].

Induction of autophagy was found significantly enhanced in U-87 MG cells following incubation with D11 for 24 and 48 h, respectively. At presence, it is not possible to assert whether induction of autophagy constitutes a stress adaptation conferring cytoprotection or an alternative cell death mechanism. However, the fact that PARP cleavage becomes visible after 48 h of incubation with D11 does not exclude that autophagy might develop as a primary response to stress stimuli in the first 24 h of incubation with the inhibitor and triggers apoptosis to kill cancer cells following a longer incubation time. This sequence of events has been shown to occur in the case of CD4/CXCR4-expressing T cells after binding of HIV-1 envelope proteins [32].

The hypoxic response includes the induction of a variety of pro-angiogenic genes. Specifically, the gene expression analysis identified strong up-regulation of *ADM*, *ANGPTL4* and *VEGFA* (Table 1). Conversely, the expression of these genes was found down-regulated in cells treated with D11 under hypoxia suggesting that D11 antagonizes the transcription of pro-angiogenesis genes through mechanisms that may be dependent or not on HIF-1α expression status.

Under hypoxic conditions, cells use glycolysis as a primary mechanism of ATP production. This metabolic switch is encouraged by stabilization of HIF-1α, which induces the expression of genes involved in metabolic adaptation including glucose uptake and the glycolytic pathway. Indeed, the expression of several of the genes involved in glucose metabolism (i.e., *HK1* and *-2*, *GLUT1* and *-3*, *GYS1*, *LDHA*, *PDK1*, *PFKFB3* and *-4*) was found up-regulated under hypoxia and marginally or largely down-regulated when cells were additionally treated with D11 (Table 1). This suggests that D11 treatment severely deprives cancer cells of oxygen and nutrient supply by suppressing the oxygen-independent metabolic pathway.

There is ample evidence that changes in metabolic activity observed in cancer cells influence the intracellular pH. Glycolysis is thought to be the major mechanism responsible for lowering the pH. Evidence indicates that the activity of carbonic anhydrase (*CA9*), which converts carbon dioxide and water to carbonic acid, is strongly induced in hypoxia contributing to low pH of the tumor. High expression levels of *CA9* have been associated with poor prognosis [33]. Our results are consistent with previous studies investigating gene expression in response to hypoxia. Accordingly, the expression of *CA9* was found largely up-regulated under hypoxia (i.e., 10.768 fold-change as compared to CT_N_), however, treatment of cells with D11 resulted in a −2.924 fold-change of *CA9* with respect to CT_H_ suggesting that D11 compromises another important adaptation of tumor cells which seems to be essential for promoting tumor invasion. In this respect, it has been shown that many proteases are activated under acidic conditions promoting tumor invasion of surrounding tissue [34].

N-myc downstream-regulated gene 1 (NDRG1) is a member of the *NDRG* gene family, which is expressed ubiquitously in tissues in response to various stress conditions including cellular differentiation, tumor progression and metastasis, DNA damage and hypoxia. Expression of *NDRG1* in response to low oxygen concentrations is HIF-1α-regulated, as induction of *NDRG1* does not occur in HIF-1α^−/−^ mouse embryo fibroblasts. However, an increase in intracellular Ca^2+^ seems to be sufficient to induce NDRG1 mRNA expression in HIF-1α-deficient cells under hypoxia (reviewed in [35]). A number of research groups have shown that NDRG1 over-expression decreases tumor growth, reduces invasion and suppresses metastasis [36,37]. However, Salnikow et al., demonstrated that NDRG1 expression is dramatically increased in aggressive prostate cancer cells [38]. In our study, *NDGR1* expression was found elevated (15.150 fold-change) under hypoxia while additional incubation with D11 resulted in a −3.6 fold-change (Table 1). There appears no clear connection in the effect of Ca^2+^ and HIF-1α on *NDRG1* induction in cancer cells. Hence, it is likely that when oxygen levels decrease, *NDRG1* induction is linked to intracellular mechanisms somewhat unrelated to metastasis and cell growth yet to be elucidated.

Finally, analysis of the gene expression array also revealed that two hypoxia-responsive genes involved in cell adhesion, invasion and vascular remodeling (i.e., *ANKRD37* and *MMP9*) were found largely down-regulated in D11 treated cells. Suppression of *ANKRD37* and *MMP9* expression may explain, at least in part, D11-mediated impaired migration of glioblastoma and pancreatic cancer cells reported previously [12] and indicates that treatment of glioblastoma cells with this compound compromises cell migration and invasion by suppressing the expression of proteins that are crucial in the metastatic process.

## 4. Materials and Methods

### 4.1. Cell Culture and Hypoxia

The human glioblastoma cell line U-87 MG was purchased from the American Type Culture Collection (ATCC, Rockville, MD, USA) and cultivated at 37 °C under a 5% CO_2_ atmosphere in Dulbecco’s modified Eagle’s medium (DMEM, Invitrogen, Taastrup, Denmark) supplemented with 10% fetal bovine serum (FBS, Biochrom AG, Berlin, Germany). Cells were treated with 4-[(*E*)-(fluoren-9-ylidenehydrazinylidene)-methyl] benzoic acid (referred to as E9, [25]), D11 (both from DTP, NIH/NCI, Rockville, MD, USA) or CX-4945 (Selleck Chemicals, Houston, TX, USA) as indicated in the figure legends. Hypoxia (1% O_2_) experiments were carried out according to [39]. Where indicated, cells were treated with 100 nM bafilomycin A_1_ (Sigma-Aldrich, Brøndby, Denmark) for 6 h.

### 4.2. Radioactive Kinase Assay

The kinase activity of human recombinant CK2α^1−335^_2_β_2_ was determined in the presence of the CK2 synthetic peptide RRRADDSDDDDD (100 μM), increasing concentrations of ATP (i.e., 5, 10, 50, 100, 200, 300 and 400 μM) and inhibitor as indicated in the figure legends and essentially as described elsewhere [40]. Kinetic parameters (i.e., K_M_ and V_max_) were calculated using GraphPad Prism version 6.0 computer software (GraphPad Software Inc., San Diego, CA, USA) based on the Michaelis-Menten plots. K_I_ values were determined from the re-plots where apparent K_M_ values and apparent V_max_ values were plotted against inhibitor concentration, respectively.

### 4.3. Cell Cycle Analysis by Flow Cytometry

Propidium iodide staining of cells and flow cytometry analysis were performed essentially as previously described [41]. Autophagy was analyzed by staining cells with 1 μg/mL acridine orange (Sigma-Aldrich) for 15 min prior harvesting by trypsinization and flow cytometry analysis.

### 4.4. Preparation of Whole Cell Lysate, Western Blot Analysis and Antibodies

Western blot analysis of whole cell lysate was performed as reported in [41]. The following primary antibodies were employed in the study: rabbit monoclonal anti-NF-κB/RelA and rabbit monoclonal anti-LC3 (both from Cell Signaling Technology, Beverly, MA, USA); mouse monoclonal anti-β-actin (Sigma-Aldrich); rabbit polyclonal anti-phospho-NF-κB/p65 (S529, Abcam, Cambridge, MA, USA); mouse monoclonal anti-PARP and mouse monoclonal anti-HIF-1α (both from BD Biosciences, San Jose, CA, USA).

### 4.5. Luciferase Reporter Assay

Determination of the transcriptional activity of HIF-1α was carried out with whole cell lysate (10 μg) from cells transfected with a luciferase reporter vector (Panomics, Affymetrix, Santa Clara, CA, USA) carrying a control sequence or HIF-1α consensus element. Transfection of cells was performed with Lipofectamine 2000 according to the manufacturer’s (Invitrogen) guidelines. After 24 h from transfection, cells were added 50 μM D11 for additional 24 h before harvesting. Cells were grown under normoxia or hypoxia in the last 24 h of incubation time as indicated in the figure legends. Determination of luciferase activity was performed employing the Luciferase Assay System kit (Promega, Stockholm, Sweden) according to the manufacturer’s recommendations. Luminescence was measured with a Perkin Elmer Victor Light 1420 luminescence counter (Perkin Elmer, San Diego, CA, USA).

### 4.6. Gene Expression Analysis by Quantitative RT-PCR Array

Total RNA samples preparation from cells treated as described in the figure legends was carried out by phenol-chloroform extraction and subsequent silica-membrane-based purification in combination with on-column DNAse digestion with the miRNeasy kit (Qiagen, Hilden, Germany) following the manufacturer’s instructions. RNA integrity was assessed by agarose gel analysis. The cDNA was prepared using the RT^2^ First strand kit (Qiagen). The expression analysis of 84 genes associated with hypoxia was carried out with the Qiagen RT^2^ Profiler PCR array according to the manufacturer’s guidelines in 96-well plates with a StepOnePlus^TM^ real-time cycler (Applied Biosystems, Nærum, Denmark). Data were normalized to five housekeeping genes included in the kit. Normalized data were analyzed using the ΔΔ*C*_t_ method with the equation ΔΔ*C*_t_ = Δ*C*_t_ (experimental sample group) − Δ*C*_t_ (control group), and the fold-change was calculated based on ΔΔ*C*_t_ with 2^−ΔΔ*C*t^ for positive changes or with −1/2^−ΔΔ*C*t^ for negative changes [42,43].

### 4.7. Statistical Analysis

Statistical significance of differences between means of two groups was determined by the two-tailed *t*-test (student’s *t*-test). The levels of significance are indicated in the figure legends.

## 5. Conclusions

Hypoxic adaptation is a frequently occurring event in tumor growth and it is associated to a more invasive phenotype and resistance to conventional treatment. This has been unequivocally demonstrated in the case of brain cancer. Hence, the identification of new therapeutic strategies targeting HIF-1α represents an attractive alternative option to the current treatment modalities. Our data show that incubation of glioblastoma cells with D11 results in rapid HIF-1α destabilization and impaired transcriptional activity. Gene expression analysis show that reduced HIF-1α expression in response to D11 treatment is accompanied by down-regulation of genes involved in angiogenesis, glucose metabolism, pH regulation, cell adhesion and invasion. Collectively, these results suggest that the combination of D11, or more potent derivatives, with existing treatments may prove to be an effective strategy in the clinics for the cure of brain cancer in the future.

## Figures and Tables

**Table 1:** Gene expression analysis.

Protein	Gene Symbol	Fold-Change	Fold-Change
		CT_H_ vs. CT_N_	D11_H_ vs. CT_H_
Adrenomedullin	*ADM*	4.358	1.226
Angiopoietin-like 4	*ANGPTL4*	7.646	−7.888
Ankyrin repeat domain 37	*ANKRD37*	2.880	−2.106
Basic helix-loop-helix family member e40	*BHLHE40*	3.229	−2.275
Bloom syndrome, RecQ helicase-like	*BLM*	−1.904	−1.549
Bcl2 interacting protein 3	*BNIP3*	5.984	1.282
Bcl2 interacting protein 3-like	*BNIP3L*	3.364	−1.058
Carbonic anhydrase IX	*CA9*	10.768	−2.924
Cyclin G2	*CCNG2*	3.367	2.445
DNA-damage-inducible transcript 4	*DDIT4*	7.233	−4.314
Egl nine homolog 1 (*C. elegans*)	*EGLN1*	1.953	−2.032
Early growth response 1	*EGR1*	−2.474	54.951
Erythropoietin	*EPO*	2.150	1.275
ERO1-like (*S. cerevisiae*)	*ERO1A*	2.033	−1.566
Coagulation factor III	*F3*	5.802	−41.328
Glucan branching enzyme 1	*GBE1*	2.523	−2.082
Glycogen synthase	*GYS1*	2.051	−2.645
Hypoxia inducible factor 3 α subunit	*HIF3A*	3.719	−3.992
Hexokinase 2	*HK2*	3.152	1.100
Insulin-like growth factor binding protein 3	*IGFBP3*	67.054	−11.170
Lactate dehydrogenase A	*LDHA*	2.602	−2.361
Lectin, galactoside-binding, soluble, 3	*LGALS3*	2.363	−2.141
Lysyl oxidase	*LOX*	5.199	−4.730
Macrophage migration inhibitory factor	*MIF*	1.986	−1.040
Matrix Metallopeptidase 9	*MMP9*	6.044	−2.535
MAX interactor 1	*MXI1*	4.017	−1.462
N-myc downstream-regulated 1	*NDRG1*	15.150	−3.611
Ornithine decarboxylase 1	*ODC1*	−2.231	11.551
Prolyl 4-hydroxylase, α 1	*P4HA1*	3.362	1.053
Pyruvate dehydrogenase kinase	*PDK1*	2.946	−1.522
6-phosphofructo-2-kinase/fructose-2,6-bisphosphatase 3	*PFKFB3*	2.394	1.146
6-phosphofructo-2-kinase/fructose-2,6-bisphosphatase 4	*PFKFB4*	3.304	−14.057
Phosphofructokinase, liver	*PFKL*	2.452	−2.309
Placental growth factor	*PGF*	2.242	2.089
Phosphoglycerate kinase 1	*PGK1*	3.159	−1.581
Plasminogen activator, urokinase	*PLAU*	−2.926	1.674
Serpin peptidase inhibitor, clade E member 1	*SERPINE1*	1.911	1.744
Solute carrier family 16 member 3	*SLC16A3*	2.454	−4.346
Solute carrier family 2 member 1 (GLUT1)	*SLC2A1*	5.890	−1.162
Solute carrier family 2 member 3 (GLUT 3)	*SLC2A3*	2.462	2.460
Thioredoxin interacting protein	*TXNIP*	3.422	1.559
Vascular endothelial growth factor A	*VEGFA*	5.367	−11.028

CT_H_: control cells (vehicle-treated) grown under hypoxia, CT_N_: control cells (vehicle-treated) grown under normoxia, D11_H_: D11-treated cells under hypoxia.
